# Vision status of children aged 7–15 years referred from school vision screening in Norway during 2003–2013: a retrospective study

**DOI:** 10.1186/s12886-019-1178-y

**Published:** 2019-08-13

**Authors:** Helle K. Falkenberg, Trine Langaas, Ellen Svarverud

**Affiliations:** National Centre for Optics, Vision and Eye Care, Faculty of Health and Social Sciences, University of South-Eastern Norway, Hasbergs vei 36, 3616 Kongsberg, Norway

**Keywords:** Health care services, Children, Vision examination, Visual status, Refractive errors

## Abstract

**Background:**

Undetected vision problems is an important cause of reduced academic achievement, performance in everyday life and self-esteem. This receives little attention in national health care services in Norway even though most of these vision problems are easily correctable. There are no published data on how many Norwegian schoolchildren are affected by correctable vision problems*.* This study aims to determine the vision status in primary and secondary schoolchildren referred from vision screening during the 10 year period of 2003–2013.

**Methods:**

Of the 1126 children (15%) aged 7–15 years referred to the university eye clinic by the school screening program, all 782 who attended the eye clinic were included in the study. Patient records were retrospectively reviewed with regard to symptoms, refractive error, best corrected visual acuity (BCVA) of logMAR, binocular vision, ocular health and management outcomes.

**Results:**

Previously undetected vision problems were confirmed in 650 (83%) of the children. The most frequent outcomes were glasses (346) or follow-up (209), but types of treatment modalities varied with age. Mean refractive errors were hyperopic for all age groups but reduced with age (ANOVA, *p* < 0.001). Overall, 51% were hyperopic, 32% emmetropic and 17% myopic. Refractive errors did not change across the decade (linear regression, all *p* > 0.05). Mean logMAR BCVAs were better than 0.0 and improved with age (ANOVA, *p* < 0.001)*.* The most prevalent symptoms were headaches (171), near vision problems (149) and reduced distance vision (107).

**Conclusions:**

The vision screening identified children with previously undetected visual problems. This study shows that the types of visual problems varied with age and that most problems could be solved with glasses. Our results stress the importance of regular eye examinations and that vision examinations should be included in primary health care services. Furthermore, there is a need for raised awareness among parents and teaching staff regarding vision problems in children.

## Background

We live in a visual world, and the ability to see effortlessly is something most people take for granted. The visual system changes in line with the growing child’s expanding visual and behavioural world, and the majority develop good vision, enabling the child to learn, master and achieve important goals in different phases of life and to contribute to society. In fact, good vision and eye health is a prerequisite for social, educational and economic independence and success [1–3].

Undetected vision problems are a major cause of reduced performance in everyday life, academic achievement and self-esteem [3–8]. Children are dependent on acquiring knowledge and skills through visual information, both in print and digital media, and by observing others. Children spend increasingly more time with their school- and homework as their academic requirements advance. At the same time, the font sizes in printed learning materials are gradually decreased. The fast development and use of digital technology further increase the visual demands in school [9, 10].

To keep up with learning and expected academic performance, children now need to spend more time reading and accessing digital information [11]. Furthermore, from a much earlier age, children spend more time using digital devices in their spare time [10–14]. This increases the workload on the visual system, especially the ability to sustain clear vision at near for longer periods at a time. Even problems like small refractive errors and accommodation or oculomotor control deficits can cause headaches, difficulty concentrating or poor coordination and may lead to unnecessary challenges in school [15–18]. If a child avoids activities such as reading and homework, there will be serious consequences for learning and for academic and social success [2, 4, 19, 20]. This means that good vision is more important than ever and that uncorrected vision problems should be identified.

In Norway, all children aged 6–16 years have free, compulsory education provided in comprehensive schools with one of the lowest pupil-teacher ratios (9:1) reported by the Organisation for Economic Co-operation and Development (OECD) [21, 22]. Both sexes have equal opportunity to education; the average number of years of education is 12.6, and most children (96%) are enrolled in pre-primary kindergartens [23]. The number of functional illiterates is low [24], and in 2018, Norway was ranked as number one for the global Human Development Index (HDI) [23]. Primary health care in Norway is provided free of charge by the national health authorities until the age of 16 years [25]. Vision and eye health are included in the public preventative health care programme up to 5 years of age [26] in order to prevent irreversible vision loss, but vision examination is not included in school health care services.

The prevalence of visual problems in primary and secondary schoolchildren (6–16 years) in Norway is unknown, but a recent Norwegian study in 16- to 19-year-olds found that more than half were hyperopes [27]. It is known that common vision problems, such as refractive errors, heterophorias and accommodative disorders, may have a profound effect on learning [4, 7]. Some of these conditions may be asymptomatic, and children are often not aware of their vision problems. Therefore, children’s vision problems may not be recognised by parents or teachers. In particular, hyperopia and near vision problems may only be detected through a thorough eye examination. Importantly, common vision problems are easy and cost-efficient to correct, and eye examinations should be considered included in school health services to promote learning, social interactions, future education, employment and socioeconomic benefits [2, 8, 20, 21].

There are no published studies describing the visual status or extent of common vision problems in primary and secondary Norwegian schoolchildren. In Kongsberg, a school vision programme has existed since the 1970s as a collaboration between the municipality and the National Centre of Optics, Vision and Eye Care (NCOVE) at the University of South-Eastern Norway. Here, all schoolchildren are offered vision screening at the ages of 7–15 years. This study aims to determine the vision status in primary and secondary school children referred from vision screening during the 10 year period of 2003–2013.

## Methods

### Study population

The annual school vision screening programme is a collaboration between the Kongsberg municipality and NCOVE and offers screening to all children in the 2nd, 5th and 10th grades (7-, 10- and 15-year-olds) in the 13 primary and secondary schools in Kongsberg. The majority of the school population has a Norwegian ethnic background (86%) similar to the rest of Norway [28]. During the decade of 2003–2013, 7658 children participated in the school vision screening programme (94% of all 8191 children eligible). Of these, 1126 (15%) children failed the vision screening and were referred to the NCOVE university eye clinic for a full eye examination.

### Study sample

**Fig. 1:**
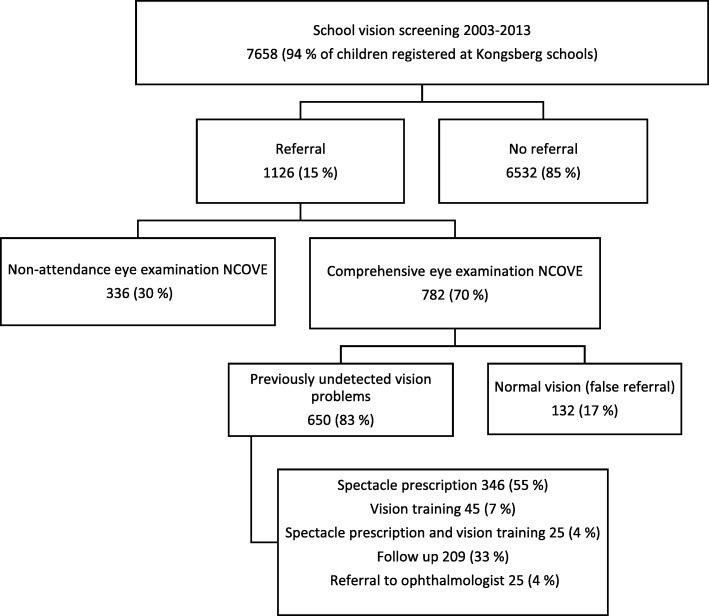
Flow chart of the visual outcome from the Kongsberg vision school screening for 2nd-, 5th- and 10th-grade children during the period 2003–2013. In each box, the number and percentage of children are reported

This study has retrospectively analysed data from the patient records of those 782 children who attended the NCOVE eye clinic. Several cohorts were screened two or three times, but only 40 (5%) children were referred more than once. See flow chart for details (Fig. 1). The study followed the tenets of the Declaration of Helsinki and was approved by the Ombudsman for Privacy in Research at the Norwegian Social Science Data Services.

### Protocol

The patient records included a structured and age-appropriate history and symptoms interview. Reported refractive status was determined after retinoscopy and subjective refraction (at 6 m), generally obtained without cycloplegia, and best corrected visual acuity (BCVA) was recorded. Ocular alignment was assessed by cover test (at 6 m and 40 cm). Near point of convergence (NPC) and monocular and binocular accommodation amplitude (ACC) were assessed using an RAF ruler. In addition ocular adnexa, pupillary reactions, anterior and posterior segments were examined. The eye examination was performed by final-year optometry students under supervision, and the supervisor had the overall responsibility for choice of management and advice to the patient in line with the Norwegian Association of Optometry’s clinical guidelines.

For analysis, spherical equivalent refraction (SER) was calculated in dioptres (D). Refractive errors were defined as emmetropia (− 0.50 < SER < + 0.50 D), hyperopia (SER ≥ + 0.50 D), myopia (SER ≤ − 0.50 D), astigmatism (≤ − 0.75 DC) and anisometropia (≥ 1.00 D) [29, 30]. Orthophoria was defined as 2 prism dioptres (pd.) exophoria to 1 pd. esophoria for distance and 6 pd. exophoria to 0 pd. esophoria for near. Normal NPC was defined as ≤10 cm and normal ACC as ≤10 D, ≤ 9 D and ≤ 8 D for the 2nd-, 5th- and 10th-graders respectively. Thirty-four children with strabismus were excluded from the binocular vision analysis. Symptoms were grouped into six categories. For details, see Table 2.

### Management outcomes

Reported management and advice given to the patient were defined by five main categories: 1) prescription, 2) vision training, 3) follow-up, 4) referral to ophthalmologist and 5) false referrals (referrals from screening where the eye examination concluded there were no symptoms and that vision was normal) (see Table 2). Prescriptions were glasses or contact lenses. Vision training was conventional home-based convergence, accommodation or facility training. Children with asthenopia or mild non-symptomatic hyperopia were scheduled for follow-up.

### Statistical methods

One-way ANOVA and post hoc Tukey HSD tests were used to analyse differences between age groups. The threshold for statistical significance level was set at 5% (*p* < 0.05). Distributions and analyses using refractive errors include right eye only, as there were no significant differences between the right and left eyes (paired t-test, *p* > 0.05) for either age group, and SERs were normally distributed. Linear regression analyses were used to investigate refractive changes over time for each age group separately. All computations were performed using the statistical package SPSS Statistics 21 (International Business Machines, USA). Incomplete data sometimes occurred, explaining varying sample sizes for different parameters.

## Results

Of the 1126 children referred, 782 (70%) attended the eye examination, with 241 (31%), 241 (31%) and 300 (38%) children in the three age groups (2nd, 5th and 10th grades, respectively).

### Refractive error and visual acuity

Monocular and binocular spherical equivalent refractive errors (SERs) (mean [95% CI]) are shown in Table 1. For all three age groups the mean SER was skewed towards hyperopia. A one way ANOVA showed a significant difference in SER between age groups (ANOVA, *F* (2, 776) = 26.8, *p* < 0.001). Post hoc comparisons using the Tukey HSD test showed a significant reduction in hyperopia (SER) between all age groups with increased age (*p* < 0.05 for all comparisons).

**Table 1:** Mean [95% CI] refractive error, best corrected visual acuity and accommodation amplitude for 2nd-, 5th- and 10th-grade children, shown for right eye, left eye and binocular measurements

		2nd grade Mean [CI]	5th grade Mean [CI]	10th grade Mean [CI]
Refraction (SER, D)	RE	+ 0.71 [+ 0.61, +0.82]	+ 0.46 [+ 0.32, + 0.60]	+ 0.07 [− 0.05, + 0.19]
	LE	+ 0.76 [+ 0.65, +0.86]	+ 0.51 [+ 0.36, + 0.66]	+ 0.08 [− 0.03, + 0.20]
Best corrected visual acuity (logMAR)	RE	+ 0.01 [− 0.002, + 0.02]	− 0.04 [− 0.06, − 0.02]	− 0.05 [− 0.06, − 0.04]
	LE	+ 0.02 [+ 0.001, + 0.03]	− 0.04 [− 0.05, − 0.03]	− 0.06 [− 0.07, − 0.04]
	Bin	− 0.02 [− 0.04, − 0.03]	− 0.09 [− 0.10, − 0.07]	− 0.10 [− 0.11, − 0.09]
Accommodation amplitude (D)	RE	12.3 [11.7, 12.9]	11.4 [10.8, 12.0]	10.3 [9.9, 10.7]
	LE	12.4 [11.8, 13.0]	11.5 [10.9, 12.0]	10.5 [10.2, 10.9]
	Bin	14.0 [13.4, 14.6]	12.9 [12.3, 13.5]	11.8 [11.3, 12.2]

*SER* spherical equivalent refractive error*, RE* right eye*, LE* left eye*, BIN* binocular*, D* dioptre

Table 2 shows that one third of the children were classified as emmetropic (33%) and slightly more than one third as low-grade hyperopic (≥ + 0.50D, < + 2.00D, 38%). Hyperopia ≥ + 2.00 D decreased with age and was present in 7% of the 2nd-grade, 6% of the 5th-grade and 1% of the 10th-grade children. Myopia increased with age, with 3% of 2nd-grade, 15% of 5th-grade and 27% of the 10th-grade children being myopic. Although almost one third of the 10th-grade children were classified as myopic, only 1% had myopia of − 3.00 D or higher. Clinically significant astigmatism (≥ 0.75 DC) was present in 5% of all children and clinically significant anisometropia (≥ 1.00 D) in 3%*.*

**Table 2:** Sample description of demographics, eye examination results, self-reported symptoms and management (*n* = 782)

		All *n* = 782 *n* (valid %)	2nd grade *n* = 241 (31) *n* (valid %)	5th grade *n* = 241 (31) *n* (valid %)	10th grade *n* = 300 (38) *n* (valid %)
Referred	Males	336 (43)	107 (44)	103 (43)	128 (43)
	Females	446 (57)	136 (56)	138 (57)	172 (57)
Ametropia RE (SER, D)	Emmetropia (> −0.50 D, < + 0.50 D)	249 (32)	76 (32)	73 (30)	100 (33)
	Hyperopia (≥ + 0.50 D, < + 2.00 D)	371 (47)	137 (57)	119 (49)	115 (38)
	Hyperopia (≥ + 2.00 D)	34 (4)	17 (7)	13 (6)	4 (1)
	Myopia (≤ − 0.50 D)	125 (16)	8 (3)	36 (15)	81 (27)
	Myopia (≤ − 3.00 D)	5 (1)	0	1 (0)	4 (1)
	Anisometropia (≥ 1.00 D)	13 (2)	1 (0)	6 (2)	6 (2)
	Astigmatism (≥ − 0.75 DC)	40 (5)	12 (5)	10 (4)	18 (6)
Best corrected visual acuity, BCVA RE	LogMAR (≤ 0.0)	699 (91)	191 (82)	220 (94)	288 (96)
	LogMAR (≤ 0.1, > 0.0)	50 (7)	32 (14)	13 (6)	5 (2)
	LogMAR (< 0.5, > 0.1)	17 (2)	10 (4)	1	6 (2)
	LogMAR (≥ 0.5)	1	1	0	0
Horizontal heterophoria distance (6 m)	Orthophoria	579 (78)	168 (74)	180 (80)	231 (82)
	Exophoria > 2 pd.	94 (13)	46 (20)	33 (15)	27 (9)
	Esophoria > 1 pd.	51 (7)	14 (6)	11 (5)	26 (9)
Horizontal heterophoria near (40 cm)	Orthophoria	563 (77)	171 (75)	174 (78)	218 (76)
	Exophoria > 6 pd.	100 (14)	33 (14)	22 (10)	45 (16)
	Esophoria > 0 pd.	74 (9)	24 (10)	27 (12)	23 (8)
Vertical heterophoria	Present > 3 pd.	5 (0)	0	2 (0)	3 (1)
Strabismus	Present	34 (4)	13 (5)	1 (0)	20 (7)
Near point of convergence, NPC (cm)	≤ 10 cm	640 (82)	200 (88)	196 (85)	240 (82)
	> 10 < 25 cm	78 (10)	14 (6)	24 (10)	40 (14)
	≥ 25 cm	34 (4)	13 (6)	10 (4)	11 (4)
Accommodation amplitude (D)	Reduced ACC*				
	RE	200 (26)	66 (30)	62 (27)	72 (25)
	LE	203 (26)	67 (30)	69 (30)	67 (23)
	BIN	126 (16)	41 (19)	43 (19)	42 (14)
Ocular pathology	Present	14 (2)	3 (1)	5 (2)	6 (2)
Symptoms	Headaches	171 (22)	37 (15)	59 (24)	74 (25)
	Reading and near task problems	149 (19)	41 (19)	51 (21)	57 (19)
	Reduced distance vision	107 (14)	26 (11)	28 (12)	53 (22)
	Focus change problems D/N/D**	62 (8)	5 (2)	18 (7)	39 (13)
	Tired eyes	55 (7)	10 (4)	16 (7)	29 (10)
	Double vision	4 (0)	2 (1)	2 (1)	0
Management	Spectacle prescription	346 (55)	60 (25)	116 (48)	170 (57)
	Vision training (VT)	45 (7)	10 (4)	18 (7)	17 (6)
	Prescription and VT	25 (4)	3 (1)	10 (4)	12 (4)
	Follow-up	209 (33)	88 (37)	58 (24)	63 (21)
	Referral ophthalmologist	25 (4)	14 (6)	5 (2)	6 (2)
	False referral	132 (17)	66 (27)	34 (14)	32 (11)

*Criteria for reduced ACC ≤ 10 D, ≤ 9 D, ≤ 8 D for 2nd-, 5th- and 10th-grade children, respectively. *RE* right eye, *LE* left eye, *BIN* binocular

**D/N/D: changes in focus from distance to near, or near to distance

**Fig. 2:**
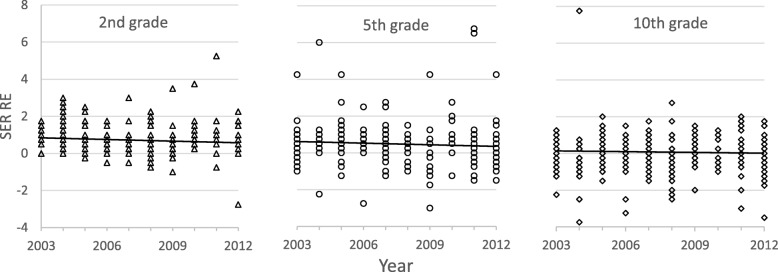
Scatterplot showing right-eye spherical refractive errors (SER RE) in dioptres (D) for each age group (2nd, 5th, and 10th grades) as a function of time for each year in 2003–2013. Points show individual children (several overlap), and lines show fitted linear regression lines

The distribution of refractive errors was similar for all three age groups over the 10-year period (Fig. 2). Linear regression shows no significant change in either myopia or hyperopia over the decade for the 2nd-grade (*R*^*2*^ = 0.01, *F* = 2.9, *p* > 0.05), 5th-grade (*R*^*2*^ = 0.01, *F* = 1.4, *p* > 0.05) or 10th-grade children (*R*^*2*^ = 0.002, *F* = 0.45, *p* > 0.05).

Most children obtained very good correctable vision (BCVA, Table 1). As expected, binocular BCVA was slightly better than monocular. Overall, 91% of the children had BCVA of logMAR 0.0 (decimal acuity 1.0) or better in one or both eyes (Table 2). Table 2 shows there was a slight but significant improvement with age in RE BCVA (ANOVA, *F* (2, 772) = 28.2, *p* < 0.001) and binocular BCVA (ANOVA, *F* (2, 766) = 44.8, *p* < 0.001). Tukey HSD post hoc analysis showed that 2nd-graders had lower RE and binocular visual acuity than both older age groups (*p* < 0.01 for all comparisons), but there was no significant difference between 5th- and 10th-graders (*p* > 0.1). Reduced vision was found in 2% of the children, including one 2nd-grade child with BCVA > logMAR 0.5 (decimal acuity < 0.3), who was referred to an ophthalmologist due to ocular pathology.

### Binocular vision and accommodation

Table 2 shows that distance and near horizontal orthophoria were present in 78 and 77% of children, respectively. The mean [95% CI] heterophoria was 0.8 [0.6, 1.0] exophoria for distance, and 2.3 [2.0, 2.6] exophoria for near. Exophoria was present in 13 and 14% of the children for distance and near, respectively (see Table 2 for criteria). Esophoria was less common and present in 7 and 9% of children for distance and near, respectively. Mean [95% CI] binocular accommodation was 14.0 [13.4, 14.6] D, 12.9 [12.3, 13.5] D, and 11.8 [11.3, 12.2] D for 2nd-, 5th- and 10th-graders, respectively (Table 1), and reduced binocular accommodation was found in 15% of all children (Table 2). ANOVA and post hoc analyses showed a significant difference in accommodation between the three age groups for binocular measures (ANOVA, *F* (2, 774) = 18.9, *p* < 0.001; Tukey HSD, *p* < 0.007 for all comparisons). NPC was 8.2 [7.7, 8.7] cm across all children, and NPC ≥ 10 cm was found in 19%. There was no significant difference between the age groups for NPC (ANOVA, *F* (2, 774) = 0.241, *p* = 0.786).

### Symptoms

Of the 782 children, 26% experienced symptoms of vision problems, and 25% had more than one symptom. The most prevalent symptoms were headaches (22%), near vision problems (19%) and reduced distance vision (14%).

### Management

Most children (650, 83%) referred from the school screening had vision problems requiring treatment or follow-up. Glasses (spectacle prescription) for distance or near work was the most common management strategy (55%), followed by vision training (7%) and glasses combined with vision training (4%). Glasses were recommended primarily for low hyperopia in 2nd- and 5th-grade children and for myopia in 10th-grade children. Binocular vision and near problems due to reduced accommodation or poor NPC were prescribed low plus lenses, vision training or both. Of the 16% of children with reduced accommodation, 42% were given glasses, 19% were prescribed vision training and 11% a combination. Of the 14% of children with reduced NPC, 40% were given glasses, 31% vision training and 20% both glasses and training. Vision training was more commonly recommended for 5th- and 10th-grade children, while the 2nd-grade children were more likely to receive follow-up. Follow-up (33%) was recommended when the child had very slight symptoms, refractive errors or binocular problems and when no immediate management was required. Overall 25 (4%) children were referred to an ophthalmologist; however, most were 2nd-grade children requiring glasses to be covered by the National Insurance Scheme.

## Discussion

This study describes vision status based on a comprehensive eye examination in 782 of 1126 (70%) children referred from the Kongsberg vision screening programme, for which 7658 2nd-, 5th- and 10th-grade children attended during 2003–2013. Most children had normal functional vision and eye health. As expected, there was a slight but significant improvement in BCVA with age, and only a few children had reduced visual acuity. However, of those 782 attending the eye examination, 83% were confirmed to be true referrals, indicating that the vision screening programme identified vision problems previously not detected. This suggests that many children, parents and teachers are unaware of vision problems that may influence academic performance and quality of life, and supports previous studies [2, 4, 15–17, 20, 31, 32].

Importantly, our study confirms that most vision problems can easily be managed by glasses or vision training [31–33]. A substantial proportion of the children had low to moderate hyperopia or accommodative or binocular deficits. These deficits may interfere with the ability to do sustained near work [17, 18, 34, 35] but can be difficult to detect because of normal distance vision and absence of explicit symptoms. As children spend a considerable amount of time on near activities at school and in their spare time [10, 13], eye and vision examinations should be available through the school health care services to prevent unnecessary academic achievement gaps.

Even though one third of children in this study were emmetropic, all age groups showed a slight hyperopic mean refractive error which reduced with age, as expected [36–40]. Interestingly, we did not find any change in refractive errors during the 10-year period for either age group, nor did we find the high proportion of myopia reported in many studies [41–45]. Although we found that myopia increased with age, only four children (1%) had myopia > 3.0 D (SER). The relatively low proportion of myopia supports previous studies showing that hyperopia is frequent in Nordic children and youths [27, 46, 47] and implies that refractive status has been relatively stable over the past few decades. This is also similar to data reported in white American children [40, 48]. One limitation to the reported refractive errors in this study is that cycloplegic refraction was not routinely used, as this was not considered standard clinical practice for schoolchildren in Norway until 2015. However, a cycloplegic refraction would skew the refractive error for all age groups towards higher values of hyperopia, and as such, the values and numbers of hyperopia in our study would increase.

Most children had good accommodation and binocular vision, but a proportion were given glasses or vision training. Accommodation and binocular vision problems may cause symptoms like headache, eyestrain, blurred vision, intermittent diplopia, poor concentration and comprehension when performing near tasks [18, 35, 49–51]. However, children do not necessarily complain of symptoms if not asked specifically. It is likely that a substantial portion of the children found to have vision problems in this study would have remained undiagnosed in absence of the vison screening.

Our results show that children in different age groups have different visual challenges that need different treatments. Academic achievements and success depend on effective and efficient reading and learning. Vision changes along with increased visual demands in school with age, and eye examinations at regular intervals should be emphasised. This study suggests that children would benefit from a comprehensive vision examination during the 2nd, 5th and 10th grades at school, as this is likely to promote academic success and social inclusion. Furthermore, it would educate and raise awareness among children, parents and teaching staff of the importance of good vision for good health.

A strength of this study is the large stable number of children attending the eye examination. The vision problems remained similar over the decade, which limits the effect of bias and contributes to the validity of this study. Furthermore, the Kongsberg school population has a Norwegian ethnic background similar to the rest of Norway, and the school vision screening has a very high attendance (94% of the population). This strengthens the potential generalisability of the study results to all Norwegian schoolchildren of the same age. A limitation of the study is the large amount of data collected over a long period by different optometry students, which may have influenced the quality of results and missing data, but in all cases, they were supervised and managed by experienced optometrists, and the results are comparable to other Nordic countries. The study does not include data after 2013 due to a time-limited approval of the study period. Furthermore, changes were made to the school vision screening protocol based on preliminary results from this study (e.g., include cycloplegic refraction). Based on the results from a recent study in Norwegian adolescents [27], we think our results would not be significantly different if more recent years were added. Despite not including recent years, our study contributes important knowledge of vision status and problems in schoolchildren that may guide further research, clinical practice and health care policies to include vision in school health care, as this still receives little attention in Norway and in many other countries [52, 53].

## Conclusions

Even though most children attending the eye clinic had good vision, at least 11% of the population screened during 2003–2013 had previously undetected vision problems. This study shows that the types of visual problems vary with age and, importantly, that most problems were solved with glasses or vision training at a low cost. Our results stress the importance of regular eye examinations and that vision examinations should be included in primary health care services to reduce the number of schoolchildren with avoidable vision problems and potential socioeconomic burden. Furthermore, there is a need for raised awareness among parents and teaching staff on vision problems in children.

## Data Availability

The datasets analysed during the current study are available from HKF on reasonable request.

## Abbreviations

ACC: Accommodation
BCVA: Best corrected visual acuity
BIN: Binocular
cm: Centimetre
D: Dioptres
LE: Left eye
m: Metre
MAR: Minimum angle of resolution
NCOVE: National Centre for Optics, Vision and Eye Care
NPC: Near point of convergence
pd.: Prism dioptres
RE: Right eye
SER: Spherical equivalent refraction
VT: Vision training
